# ICTV Virus Taxonomy Profile: Peribunyaviridae 2024

**DOI:** 10.1099/jgv.0.002034

**Published:** 2024-11-07

**Authors:** William M. de Souza, Charles H. Calisher, Jean Paul Carrera, Holly R. Hughes, Marcio R. T. Nunes, Brandy Russell, Natasha L. Tilson-Lunel, Marietjie Venter, Han Xia

**Affiliations:** 1 University of Kentucky, Lexington, KY, USA; 2 Colorado State University, Fort Collins, CO, USA; 3 Instituto Conmemorativo Gorgas de Estudios de la Salud, Panama City, Panama; 4 Centers for Disease Control and Prevention, Fort Collins, CO, USA; 5 Universidade Federal do Pará, Bélem, Brazil; 6 Indiana University, Indianapolis, USA; 7 University of the Witwatersrand, Johannesburg, South Africa; 8 Wuhan Institute of Virology, Wuhan, PR China

**Keywords:** bunyavirus, ICTV Report, *Orthobunyavirus*, *Peribunyaviridae*, taxonomy

## Abstract

Peribunyavirids produce enveloped virions with three negative-sense RNA segments comprising 10.7–12.5 kb in total. The family includes globally distributed viruses in multiple genera. While most peribunyavirids are maintained in geographically restricted vertebrate–arthropod transmission cycles, others are arthropod-specific or do not have a known vector. Arthropods can be persistently infected. Human and other vertebrate animal infections occur through blood feeding by an infected vector arthropod, resulting in diverse human and veterinary clinical outcomes in a strain-specific manner. Reassortment can occur between members of the same genus. This is a summary of the International Committee on Taxonomy of Viruses (ICTV) Report on the family *Peribunyaviridae*, which is available at ictv.global/report/peribunyaviridae.

## Virion

**Fig. 1.**
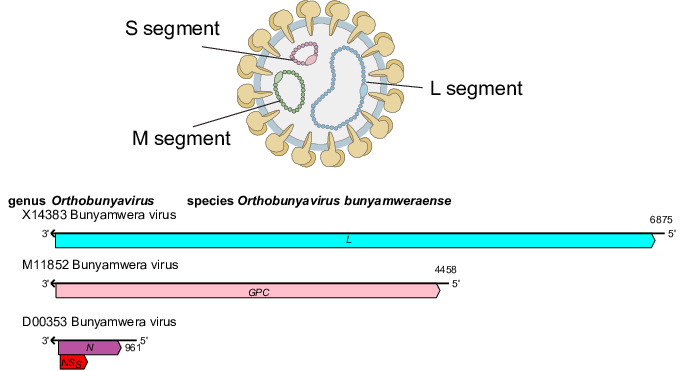
*Peribunyaviridae* family. Representation of a virion in cross-section (top). Generalized coding strategy of a peribunyavirid (bottom).

Peribunyavirids produce virions that are spherical or pleomorphic, 80–120 nm in diameter [1], with glycoprotein surface projections (5–18 nm) embedded in a lipid bilayer envelope (about 5 nm) (Table 1, Fig. 1). Ribonucleoprotein complexes are associated with the L protein and are made of non-covalently closed, circular genomic RNAs that are individually encapsidated [2].

**Table 1.** Characteristics of members of the family *Peribunyaviridae*

Example	Bunyamwera virus (S, D00353; M, M11852 and L, X14383), species *Orthobunyavirus bunyamweraense*, genus *Orthobunyavirus*
Virion	Enveloped, spherical or pleomorphic virions, 80–120 nm in diameter
Genome	Three single-stranded, negative-sense RNA molecules, small (S: 0.7–1 kb), medium (M: 2.6–4.6 kb) and large (L: 6.6–7.4 kb)
Replication	Cytoplasmic; primary transcription is primed by ‘cap snatching’ of host RNAs
Translation	On ER-bound ribosomes for G_N_ and G_C_ and on free ribosomes in the cytoplasm for N and L
Host range	Vertebrates and invertebrates (including mammals, birds, fish, mosquitoes, culicoids and psychodid sandflies)
Taxonomy	Realm *Riboviria*, kingdom *Orthornavirae*, phylum *Negarnaviricota*, subphylum *Polyploviricotina*, class *Bunyaviricetes*, order *Elliovirales*: the family includes ≥8 genera and ≥148 species

## Genome

Peribunyavirid genomes comprise three single-stranded negative-sense RNAs (designated S, small; M, medium and L, large) (Fig. 1), each with complementary terminal nucleotide sequences that base pair to form non-covalently closed, circular genomic RNAs. The S segment encodes the nucleoprotein (N), which is abundant in infected cells and facilitates genomic RNA encapsidation and virus replication. In some viruses, an overlapping reading frame encodes the non-structural protein NS_S_ [3]. The M segment encodes two structural glycoproteins (G_N_ and G_C_) responsible for viral entry into host cells. Some members also encode a non-structural protein (NS_M_) between the G_N_ and G_C_ coding regions [4]. The L segment encodes the L protein, which has RNA-directed RNA polymerase and endonuclease functions essential for viral replication.

## Replication

Virions attach via surface glycoproteins, entering the cell through clathrin-mediated endocytosis. Fusion of the viral G_C_ protein fusion peptide with endosomal membranes facilitates the release of ribonucleocapsids into the cytoplasm. The complementary 5′- and 3′-termini are promoters for mRNA and antigenome synthesis. Viral mRNAs are not polyadenylated and are truncated relative to the viral (genomic) RNA (vRNA); a 5′-methylated cap is derived from host mRNA via ‘cap snatching’ mediated by the endonuclease function of the L protein. Proteins are translated on free ribosomes (S and L segment mRNAs) or membrane-bound ribosomes (M segment mRNA). The G_N_ and G_C_ proteins are generated by co-translational cleavage and are targeted to and retained in the Golgi complex. Ribonucleoproteins are targeted near the Golgi complex. Genomes are packaged by signals from non-conserved sequences in the terminal untranslated regions. Virions bud into Golgi cisternae and are transported to the cell surface by the secretory pathway [5].

## Taxonomy

**Fig. 2.**
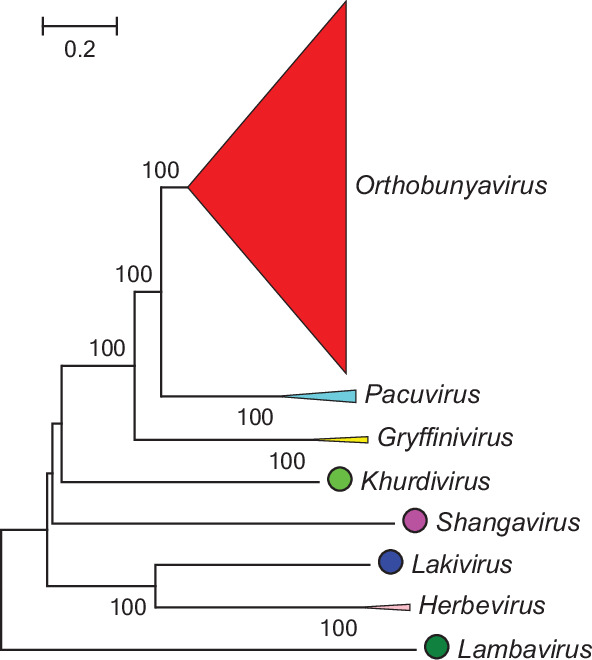
Phylogenetic analysis of the L protein of peribunyavirids.

Current taxonomy: ictv.global/taxonomy. Genera are monophyletic based on the analysis of the virus L protein (Fig. 2); members of a genus have similar genomic organizations and transmission cycles. Peribunyavirids share some of the following characteristics: (i) enveloped spherical or pleomorphic virions; (ii) three segments of single-stranded, negative-sense RNA, with all proteins encoded in the same sense and (iii) capped but not polyadenylated viral mRNA.

## Resources

Full ICTV Report on the family *Peribunyaviridae*: ictv.global/report/peribunyaviridae.

## Abbreviations

ICTV: International Committee on Taxonomy of Viruses
